# Characterization of T-Cell Responses to SMX and SMX-NO in Co-Trimoxazole Hypersensitivity Patients Expressing HLA-B*13:01

**DOI:** 10.3389/fimmu.2021.658593

**Published:** 2021-04-29

**Authors:** Jirawat Pratoomwun, Paul Thomson, Kanoot Jaruthamsophon, Rawiporn Tiyasirichokchai, Pimonpan Jinda, Ticha Rerkpattanapipat, Wichittra Tassaneeyakul, Nontaya Nakkam, Pawinee Rerknimitr, Jettanong Klaewsongkram, Yuttana Srinoulprasert, Munir Pirmohamed, Dean J. Naisbitt, Chonlaphat Sukasem

**Affiliations:** ^1^ Division of Pharmacogenomics and Personalized Medicine, Department of Pathology, Faculty of Medicine Ramathibodi Hospital, Mahidol University, Bangkok, Thailand; ^2^ Department of Clinical Chemistry, Faculty of Medical Technology, Huachiew Chalermprakiet University, Samut Prakan, Thailand; ^3^ MRC Centre for Drug Safety Science, Department of Molecular and Clinical Pharmacology, University of Liverpool, Liverpool, United Kingdom; ^4^ Division of Human Genetics, Department of Pathology, Faculty of Medicine, Prince of Songkla University, Hat Yai, Thailand; ^5^ Division of Allergy Immunology and Rheumatology, Department of Medicine, Faculty of Medicine, Ramathibodi Hospital, Mahidol University, Bangkok, Thailand; ^6^ Department of Pharmacology, Faculty of Medicine, Khon Kaen University, Khon Kaen, Thailand; ^7^ Division of Dermatology, Department of Medicine, Faculty of Medicine, Skin and Allergy Research Unit, Chulalongkorn University, Bangkok, Thailand; ^8^ Skin and Allergy Research Unit, Division of Allergy and Clinical Immunology, Department of Medicine, Faculty of Medicine, Chulalongkorn University, Bangkok, Thailand; ^9^ Department of Immunology, Faculty of Medicine Siriraj Hospital, Mahidol University, Bangkok, Thailand

**Keywords:** co-trimoxazole, drug hypersensitivity, human leukocyte antigen, sulfamethoxazole, T cell

## Abstract

HLA-B*13:01-positive patients in Thailand can develop frequent co-trimoxazole hypersensitivity reactions. This study aimed to characterize drug-specific T cells from three co-trimoxazole hypersensitive patients presenting with either Stevens-Johnson syndrome or drug reaction with eosinophilia and systemic symptoms. Two of the patients carried the HLA allele of interest, namely HLA-B*13:01. Sulfamethoxazole and nitroso sulfamethoxazole specific T cell clones were generated from T cell lines of co-trimoxazole hypersensitive HLA-B*13:01-positive patients. Clones were characterized for antigen specificity and cross-reactivity with structurally related compounds by measuring proliferation and cytokine release. Surface marker expression was characterized *via* flow cytometry. Mechanistic studies were conducted to assess pathways of T cell activation in response to antigen stimulation. Peripheral blood mononuclear cells from all patients were stimulated to proliferate and secrete IFN-γ with nitroso sulfamethoxazole. All sulfamethoxazole and nitroso sulfamethoxazole specific T cell clones expressed the CD4+ phenotype and strongly secreted IL-13 as well as IFN-γ, granzyme B and IL-22. No secretion of IL-17 was observed. A number of nitroso sulfamethoxazole-specific clones cross-reacted with nitroso dapsone but not sulfamethoxazole whereas sulfamethoxazole specific clones cross-reacted with nitroso sulfamethoxazole only. The nitroso sulfamethoxazole specific clones were activated in both antigen processing-dependent and -independent manner, while sulfamethoxazole activated T cell responses *via* direct HLA binding. Furthermore, activation of nitroso sulfamethoxazole-specific, but not sulfamethoxazole-specific, clones was blocked with glutathione. Sulfamethoxazole and nitroso sulfamethoxazole specific T cell clones from hypersensitive patients were CD4+ which suggests that HLA-B*13:01 is not directly involved in the iatrogenic disease observed in co-trimoxazole hypersensitivity patients.

## Introduction

Co-trimoxazole (CTX) is a combination drug consisting of trimethoprim (TMP) and sulfamethoxazole (SMX). It is commonly used for treatment of urinary tract infections due to *E. coli*, *Klebsiella* and *Enterobacter* spp. and also suitable for gastrointestinal infections against *E. coli*, *Shigella* spp. and *Salmonella typhi*. It is the drug of choice for the treatment and prophylaxis of *Pneumocystis jirovecii* pneumonia (PCP) in the Human Immunodeficiency Virus (HIV) patients (1). Approximately 1 to 3% of CTX prescribed HIV-uninfected patients develop mild to serious skin reactions including erythema multiforme, Stevens–Johnson syndrome (SJS), toxic epidermal necrolysis (TEN) and drug rash with eosinophilia and systemic symptoms (DRESS), whereas such reactions occurred in about 40 to 80% of HIV-infected patients (2–4).

Several forms of drug hypersensitivity reactions are associated with the carriage of human leukocyte antigens (HLA). Presentation of drug, drug modified or altered peptide sequences (due to drugs binding deep within the peptide binding groove) on the surface of HLA to T cell lymphocytes may stimulate an immune response *via* triggering of the T cell receptor (TCR) (5, 6). Co-trimoxazole-induced SJS/TEN is associated with HLA-B*15:02, HLA-C*06:02, and HLA-C*08:01 in Thai population (7) and HLA-B*38 in Europeans (8). Interestingly, our previous case-control study demonstrated that HLA-B*13:01 is associated with co-trimoxazole-induced DRESS in Thai population, while co-trimoxazole-induced SJS/TEN was associated with HLA-B*15:02 (9). This observation on co-trimoxazole-induced SJS/TEN was consistent with previous studies (7). However, not all patients with an *HLA* risk allele developed reactions. As T cells are thought to be involved in the molecular pathogenesis of many forms of severe cutaneous adverse reactions (10–12), a global TCR repertoire analysis in HLA-B*15:02 positive patients with carbamazepine-induced SJS/TEN was studied and clearly demonstrated that restricted TCR usage of drug-specific T cells participated in the development of a reaction (8). Moreover, the analysis of TCR Vβ repertoire of HLA-B*57:01 positive patients susceptible to abacavir hypersensitivity illustrated polyclonal TCR usages recognize the drug-HLA complex, then driving the T cell activation (13–15).

T cell activation and the release of effector molecules depends on drug (antigen) recognition by T cell receptors located on the cell surface. The T cell receptor receives signals from the drug, peptide and HLA protein which form a complex and are displayed on the surface of antigen presenting cells. Thus, this study aimed to characterize the T cell responses from Thai patients with co-trimoxazole-induced drug rash with eosinophilia and systemic symptoms. The assessment of specific T cell responses is essential to better understand the nature of the immune response induced and disease progression.

## Materials and Methods

### Study Population

Co-trimoxazole-induced Stevens–Johnson syndrome (SJS) and drug reaction with eosinophilia and systemic symptoms (DRESS) patients were recruited from Ramathibodi Hospital and Srinagarind Hospital between 2018 and 2019. All of the patients studied were HIV negative. Reactions were assessed by two dermatologists or allergists who reviewed photographs, pathological slides, clinical morphology and medical records. SJS is defined as skin detachment of BSA < 10%, the clinical features of DRESS follow criteria from RegiSCAR and is defined as patients presenting with fever, maculopapular rash with internal organ involvement, and hematologic abnormalities. We evaluated co-trimoxazole was the causative drug of SJS or DRESS using Naranjo algorithm (16), the score of the algorithm of drug causality for epidermal necrolysis (ALDEN) (17). The cases defined as possible, probable and definite were recruited in this study. The blood samples were collected after the patients recovered from the reaction between 2-5 years. The lymphocyte transformation test (LTT) and IFN-γ ELISpot was also performed on patient’s PBMC to identify the presence of circulating drug responsive T cells.

The study was performed according to the approval by the Ramathibodi Hospital and Srinagarind Hospital ethical review board, and both informed and written consent forms were obtained from all the participants.

### DNA Extraction and *HLA* Genotyping

The DNA was extracted from PBMC by DNA extraction automated MagNA Pure Compact (Roche Diagnostics GmbH, Germany). The concentration of genomic DNA for all individuals was assessed by using NanoDrop 2000 for measuring the genomic DNA as well as purity with dynamic range around 220 to 750 nm. Wavelength at 260 nm is suitable for measuring the genomic DNA and at 280 nm was used to evaluate contaminated protein in the sample.

HLA alleles were genotyped using sequence-specific oligonucleotides (PCR-SSOs). In brief, the diluted DNA sample obtained from each patient were amplified polymerase chain reaction (PCR) by GeneAmp^®^PCR System 9700 (Applied Biosystems, Waltham, USA). The PCR product was then hybridized against a panel of oligonucleotide probes on coated polystyrene microspheres that had sequences complementary to stretches of polymorphism within the target HLA alleles using the Lifecodes HLA SSO typing kits (Immucor, West Avenue, Stamford, USA). The amplicon-probe complex was then visualized using a colorimetric reaction and fluorescence detection technology by the Luminex^®^IS 100 systems (Luminex Corporation, Austin, Texas, USA). Analysis of the HLA alleles was performed using MATCH IT DNA software version 3.2.1 (One Lambda, Canoga Park, CA, USA).

### Chemicals, Cell Culture, Generation of EBV

Dapsone (DDS), SMX and TMP were purchased from Sigma-Aldrich, (Buchs, Switzerland). EBV-transformed autologous B lymphoblastoid cell lines (B-LCLs) were used as antigen-presenting cells (APCs). PBMCs were isolated from co-trimoxazole induced DRESS carrying HLA-B*13:01 using Ficoll density gradient centrifugation. For APC generation, the supernatant of B95.8 cells was filtered and added to 5x10^6^ PBMCs, then 1 µg/mL cyclosporin A (CSA) was added. The PBMC were then incubated in 5% CO_2_ incubator at 37°C overnight. The mixture was centrifuged at 1500 rpm for 10 minutes, then the cells were re-suspended in 2 mL culture medium with CSA and transferred to a 24 well plate. Culture medium consisted of RPMI 1640, 10% pooled fetal bovine serum (FBS), HEPES buffer (25mM), L-glutamine (2mM), streptomycin (100 µg/mL) and penicillin (100 U/mL). To maintain B cell transformation, medium and CSA were replaced twice a week for 3 weeks. Eventually the transformed B cell lines were maintained with medium in the absence of CSA before being transferred to a flask. These cells were used as a ready supply of immortalized autologous APC.

### Generation and Characterization of Drug Specific T Cell Clones

T cell lines were generated by culturing PBMCs with dapsone (DDS, 125 µM), nitroso-dapsone (DDS-NO, 10 µM), sulfamethoxazole (SMX, 1 mM) and nitroso-sulfamethoxazole (SMX-NO, 20 µM) in medium for 14 days (37°C; 5% CO_2_) and media containing IL-2 (2 µL/mL) was added to maintain proliferation on day 6 and 9. Culture medium consisted of RPMI 1640, 10% pooled heat inactivated human AB serum, HEPES buffer (25 mM), L-glutamine (2 mM), transferrin (25 µg/mL), streptomycin (100 µg/mL), penicillin (100 U/mL). T cells clones were generated by serial dilution (18). The characterization of T cell clones was conducted in terms of cellular surface marker expression, HLA mismatch assay, HLA restriction assay, antigen presenting cell pulsing and fixation assay, the effect of glutathione and enzyme inhibitor; methimazole (an inhibitor of peroxidases and flavin–mono-oxygenases; Meth) and 1-aminobenzotriazole (a nonselective suicide inhibitor; ABT). Detailed methods are provided as **Supplementary S1** and **Supplementary Figure 1**.

## Results

### Clinical Manifestation of Patients and *In Vitro* Activation of Patient’s Peripheral Blood Mononuclear Cells

Three patients that developed CTX-induced SJS and DRESS were utilized in this study. The causality assessment and *in vitro* test of the patients are shown in **Table 1**. PBMC from all patients were stimulated to proliferate and secrete IFN-γ with SMX-NO. PBMC from one patient were also stimulated with SMX, the parent compound. Additionally, PBMC from all patients secreted IFN-γ once PBMC were cultured with SMX. The proliferation of PMBC from one patient was observed when PBMC were cultured with nitroso-dapsone (DDS-NO), a structurally-related compound (**Supplementary Figure 2**).

**Table 1:** Clinical characteristics, causality assessment and *in vitro* test of the patients with co-trimoxazole-induced SCARs.

Patient ID	Sex	Age	Clinicalmanifestation	Onset of reaction (days)	SCARs	LTT	IFN-γ ELISpot	Naranjo score		Alden score		DRESS score	
								Score	Remark	Score	Remark	Score	Remark
BAC-02	Male	27	Maculopapular rash: face and extremities, abnormal liver function tests	28	DRESS	+	+	6	Probable	N/A	N/A	5	Probable
BAC-08	Female	25	Generalized dusky erythematous patches with some vesicles on neck with nikolsky’s sign on neck and upper chest	9	SJS	+	+	5	Probable	4	Probable	N/A	N/A
BAC-12	Female	44	Confluent maculopapular rash on trunk and extremities, abnormal liver function tests	30	DRESS	+	+	6	Probable	N/A	N/A	4	Probable

DRESS, drug reaction with eosinophilia and systemic symptoms; ELISpot, enzyme-linked immunospot; IFN-γ, Interferon gamma; LTT, lymphocyte transformation test; N/A, not available; SCARs, severe cutaneous adverse reactions; SJS, Stevens-Johnson syndrome.

### Generation and Characterization of Drug Specific T Cell Clones

For BAC-02, two of forty-nine clones and three of sixty-four clones were specific to SMX and SMX-NO, respectively. For BAC-12, eight of thirty-two clones were specific to SMX-NO. No specific clones were generated from BAC-08. Cellular surface marker expression was assayed using flow cytometry. All SMX and SMX-NO specific T cell clones expressed the CD4+ phenotype as shown in **Table 2**.

**Table 2:** Number, cellular phenotype and cross reactivity of drug-specific T cell clones.

Patients	Total number of clones	Number of specific clones	Phenotype (%)	Cross reactivity (%)			
			CD4	SMX	SMX-NO	DDS	DDS-NO
BAC-02							
- SMX	49	2	100	0	50	0	0
- SMX-NO	64	3	100	0	0	0	0
BAC-08							
- SMX	9	0	–	–	–	–	–
- SMX-NO	47	0	–	–	–	–	–
BAC-12							
- SMX	1	0		0	0	0	0
- SMX-NO	32	8	100	0	0	0	25

DDS, dapsone; DDS-NO, nitroso dapsone; SMX, sulfamethoxazole; SMX-NO, nitroso sulfamethoxazole.

-, not done.

**Figure 1:**
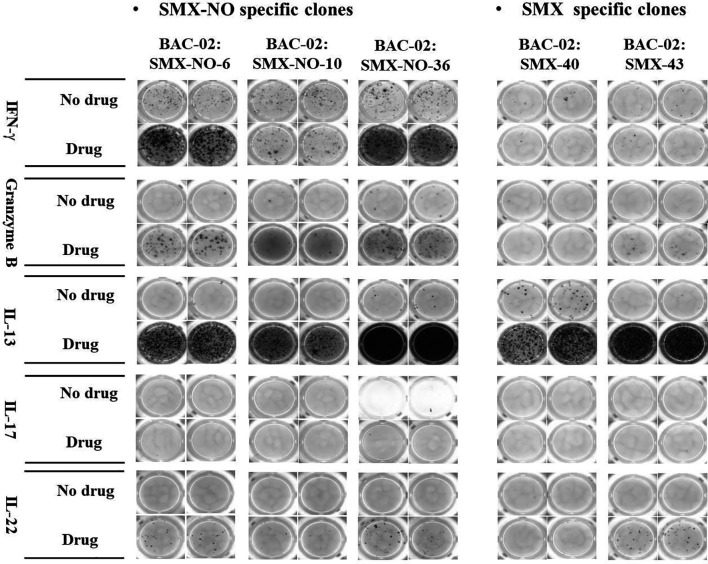
ELISpot images of cytokine secretion by SMX-NO and SMX specific T cell clones. TCCs (5x10^4^) were cultured with irradiated autologous EBV-transformed B-cells (1x10^4^) in the presence or absence of SMX-NO (40 µM) or SMX (1 mM) in an ELISpot plate pre-coated for IFN-γ, granzyme B, IL-13, IL-17 and IL-22 for 48h (37˚C; 5% CO_2_). Following incubation, the plate was developed according to the manufactures instructions visualized by ELISpot AID reader.

Twenty five percent of SMX-NO specific clones displayed cross-reactivity with DDS-NO, a structurally-related drug metabolite, but not SMX, whereas SMX specific clones cross-reacted with SMX-NO only (**Table 2** and **Supplementary Figure 3**). High levels of IL-13 were detected from all T cell clones, while some T cell clones weakly secreted IFN-γ, granzyme B and IL-22. Interestingly, no T cell clones secreted IL-17 (**Figure 1**).

### Activation of CD4+ Clones With SMX and SMX-NO is HLA Class II Restricted

**Figure 2:**
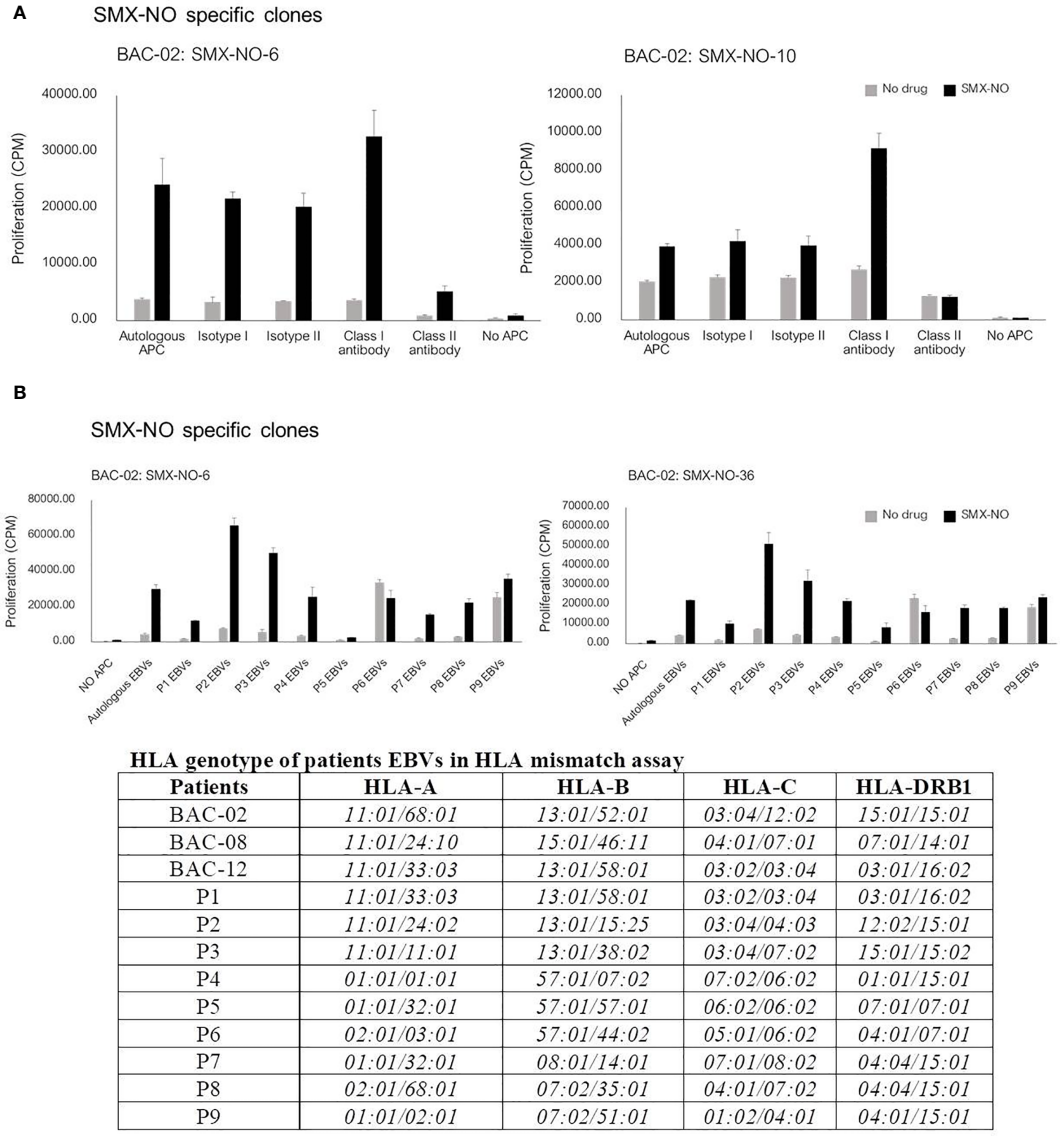
The proliferative response of SMX-NO specific T cell clones. **(A)** T cell clones (5x10^4^) were culture with autologous EBV-transformed B-cells (1x10^4^) and SMX-NO (40 µM) in the presence or absence of HLA class I and class II blocking antibodies for 48 hours (37°C, 5% CO_2_). Following incubation, [3H]-thymidine (0.5 μCi) were added to measure proliferative response. **(B)** T cell activation of SMX-NO clones in the response of different HLA-B. T cell clones (5x10^4^) were cultured with SMX-NO (40 µM) and irradiated EBV-transformed B-cells (1x10^4^) from 9 different patients carrying HLA-B*13:01 (P1-3), -B*57:01 (P4-6) and other *HLA-B* (P7-9).

The proliferation of T cell clones to SMX-NO was blocked in the presence of HLA class II blocking antibody (**Figure 2A**), indicating that the proliferative response of CD4+ specific T cells might be HLA class II restricted. Additionally, to investigate the involvement of HLA-B*13:01 in the co-trimoxazole hypersensitivity reaction, SMX-NO specific T cell clones were cultured with EBV-transformed B cells from three other patients carrying HLA-B*13:01 (P1-3), three patients EBVs carrying HLA-B*57:01 (P4-6) and cells from three donors carrying other alleles (not HLA-B*13:01 or -B*57:01, P7-9). **Figure 2B** shows T cell clones were stimulate to proliferate in the presence of SMX-NO and antigen presenting cell expressing a range of HLA class I and II molecules.

### SMX-NO Binds Covalently to Antigen Presenting Cells and Activates CD4+ T Cells Through Processing-Dependent and Processing-Independent Manners

**Figure 3:**
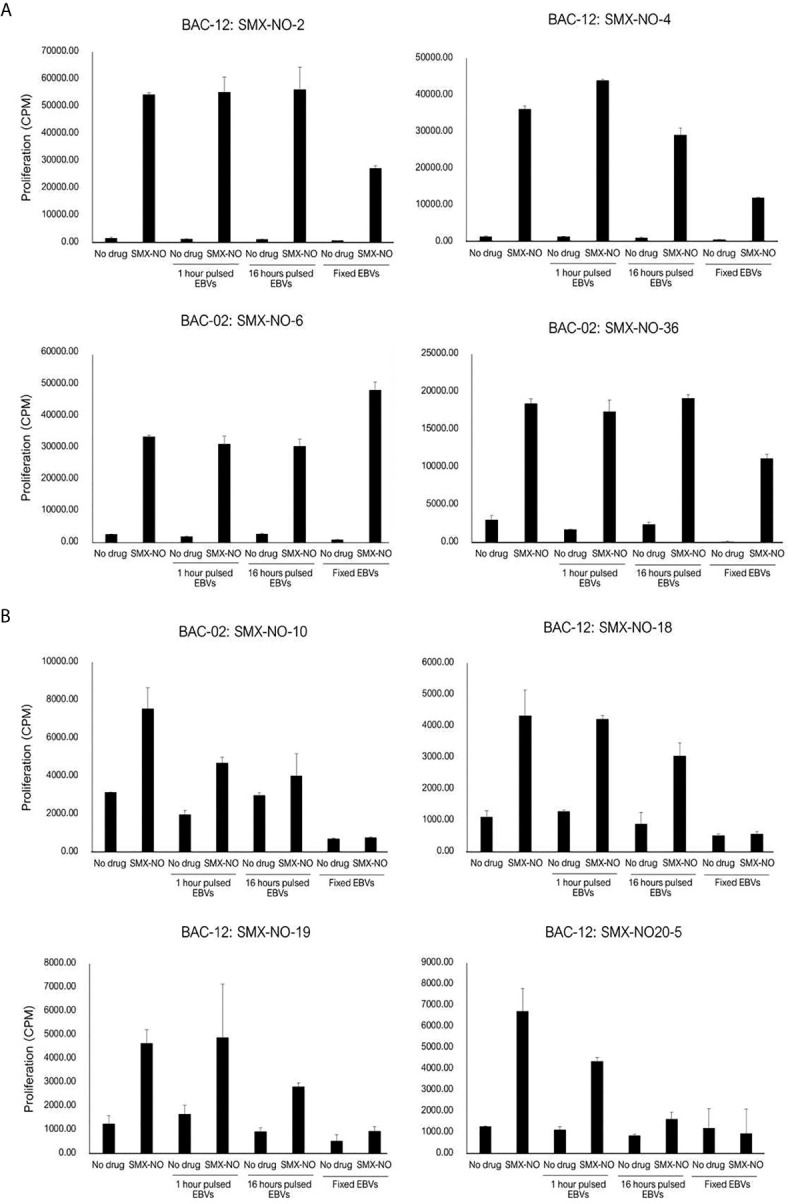
SMX-NO stimulates specific T cell *via* antigen processing-dependent and processing-independent pathways. Autologous EBV-transformed B-cells (1x10^4^) were incubated with T cell clones (5x10^4^) in the presence or absence of SMX-NO (40 µM) for 1 and 16 hours. For fixation assay, SMX-NO specific clones (5x10^4^) were cultured with either irradiated or glutaraldehyde-fixed autologous EBV-transformed B-cell (1x10^4^) in the presence of SMX-NO (40 µM) for 48 hours (37˚C; 5% CO_2_). [^3^H]-thymidine (0.5 μCi) incorporation was used to measure proliferative response. **(A)** SMX-NO T cell clones are stimulated in the presence of glutaraldehyde-fixed APC **(B)** Glutaraldehyde-fixed APC reduced the proliferative response of SMX-NO T cell clones.

Eight SMX-NO specific clones were used to investigate pathways of drug presentation. The proliferative response of four clones was blocked when APC were fixed with glutaraldehyde. In contrast, with the other four clones, T cell proliferative responses were detected when the drug metabolite was presented by irradiated or fixed APC (**Figure 3**). All clones were stimulated to proliferate when APC pulsed with SMX-NO for 1 or 16 h were added to the assay as a source of antigen (**Figure 3**). The strength of the induced response was similar to that observed with the soluble drug metabolite.

**Figure 4:**
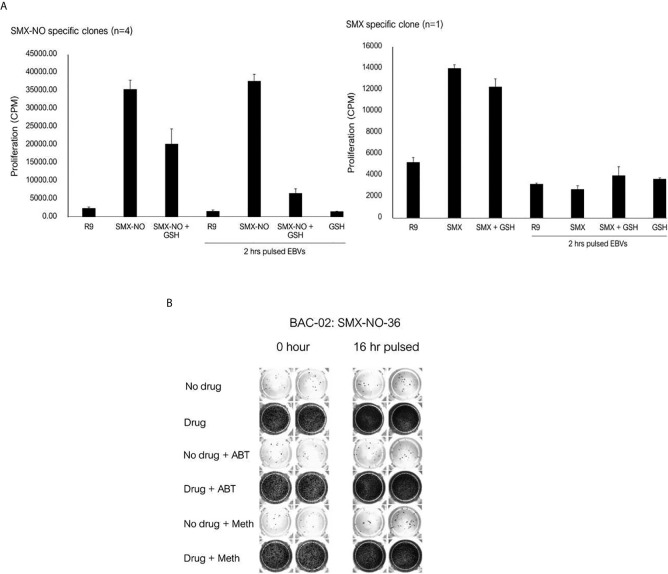
The proliferative response of SMX-NO and SMX specific T cell clones in the presence of glutathione (GSH) and enzyme inhibitors. **(A)** Autologous EBV-transformed B-cells (1x10^4^) were culture with T cell clones (5x10^4^) in the presence or absence of GSH (1 mM). For pulsing EBVs, T cell clones (5x10^4^) were culture with and without 2 h pulsed-antigen presenting cells (1x10^4^) in the presence or absence of SMX-NO (40 µM) or SMX (1 mM) for 48 hours (37˚C; 5% CO_2_). After incubation, [3H]-thymidine (0.5 μCi) were added to measure proliferative response. **(B)** 16 h-enzyme inhibitor pulsed EBVs (1x10^4^) were incubated with T cell clones (5×10^4^) for 48 hours (5% CO_2_ at 37˚C). For normal condition, Autologous EBV-transformed B-cells were cultured with T cell clones and enzyme inhibitors for 1 hour (5% CO_2_ at 37˚C) and 40 µM nitroso sulfamethoxazole. Following incubation, the plate was developed according to the manufactures instructions visualized by ELISpot AID reader. Methimazole; Meth, 1-aminobenzotriazole; ABT.

In separate experiments, the SMX-NO specific T cell clones were incubated with autologous APC and SMX-NO in the presence and absence of glutathione (GSH), which functions to reduce SMX-NO protein binding *via* quenching the metabolites reactivity. The proliferative response of SMX-NO specific clones reduced when GSH was cultured with soluble SMX-NO. Furthermore, proliferative responses were inhibited when GSH was included in a 2h APC pulsing experiments (**Figure 4A**). T cell clones abrogated by the 2 h-pulse with GSH (**Figure 4A**). Activation of SMX-NO specific clones was not altered in the presence of enzyme inhibitors ABT and methimazole (**Figure 4B**).

### SMX Specific Clones Are Activated Through a Direct HLA Binding Interaction

**Figure 5:**
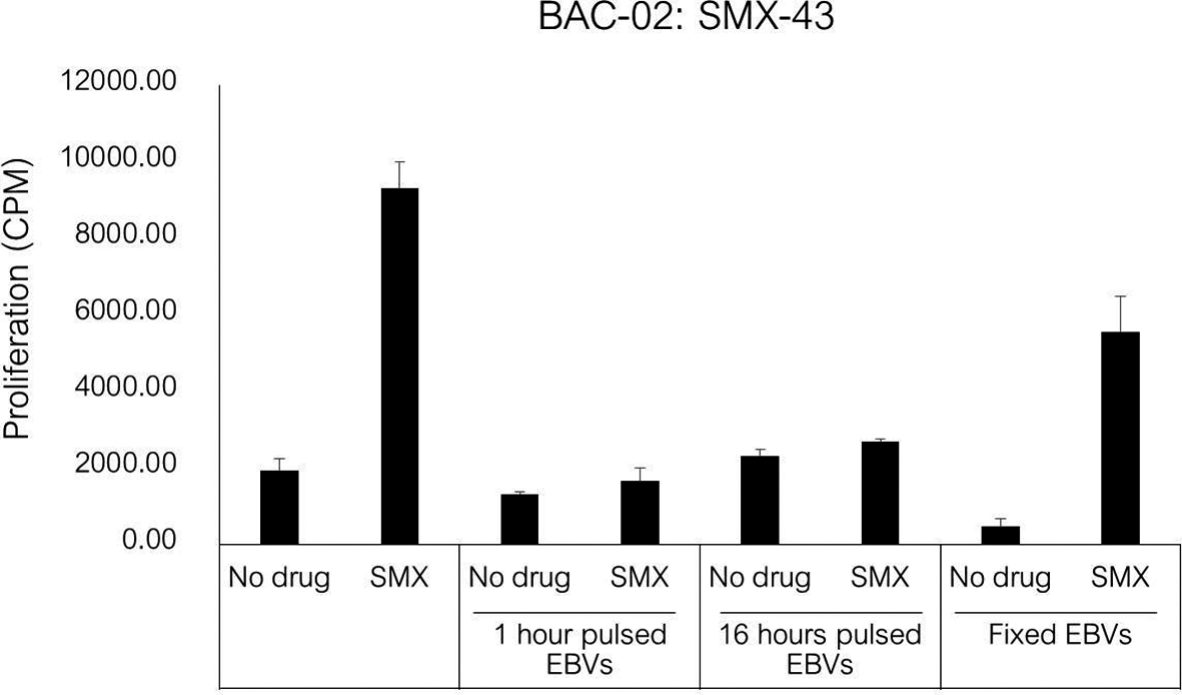
T cell activation in response to antigen stimulation of SMX specific clone. Autologous EBV-transformed B-cells (1x10^4^) were incubated with SMX (1 mM) for 1 and 16 hours, and then incubated with T cell clones (5x10^4^) after three washing steps. For fixation assay, SMX specific T cell clones (1x10^4^) were cultured with either irradiated or glutaraldehyde-fixed autologous EBV-transformed B-cell (5x10^4^) in the presence of SMX (1 mM) for 48 hours (37°C, 5% CO_2_). After incubation, [3H]-thymidine (0.5 μCi) incorporation was used to measure proliferative response.

The SMX specific T cell clone was stimulated to proliferate in the presence of soluble drug, but not with APC pulsed with SMX for 1 or 16h (**Figure 5**). The T cell proliferative response was observed when the clone was cultured with soluble SMX and glutaraldehyde-fixed APC. The presence of GSH and ABT had no effect on activation of the SMX specific T cell clone with soluble drug.

## Discussion

Several studies have shown a strong association between expression of a particular HLA allele and an increased susceptibility to drug hypersensitivity reactions. For example, HLA-B*13:01 is associated with dapsone-induced hypersensitivity reactions among leprosy patients and non-leprosy patients in Chinese and Thai (19–21). Other genetic associations between medication-induced cutaneous adverse reactions and specific *HLA* alleles have been identified in various populations, including HLA-B*57:01 and abacavir in Western Australian and North American populations (22, 23), HLA-B*15:02 and carbamazepine in Han Chinese and Thai populations (24–27), HLA-A*31:01 and carbamazepine and HLA-A*32:01 and vancomycin in European populations (28, 29) and HLA-B*58:01 and allopurinol in Han Chinese, Japanese and Thai populations (30–32). However, known associations between expression of an *HLA* risk allele and co-trimoxazole hypersensitivity are limited. Only one study by Kongpan et al. (7) demonstrated that HLA-B*15:02, HLA-C*06:02, and HLA-C*08:01 were significantly associated with co-trimoxazole-induced SJS/TEN. Recently, a genetic study showed that the HLA-B*13:01 allele is associated with co-trimoxazole-induced DRESS in Thai population (9).

In this study, T cells were characterized from co-trimoxazole hypersensitive patients to 1) define the nature of the antigenic determinant that activates T cells, 2) determine pathways of drug-specific T cell activation and 3) explore whether drug HLA-B*13:01 binding is directly involved in the T cell response. The lymphocyte transformation test is a useful tool to define the causative agent that can be performed during the recovery phase of a hypersensitivity reaction (33, 34). A small cohort of CBZ-hypersensitive patients demonstrated that the lymphocyte transformation test was positive in only the hypersensitive patients (35), while in β-lactam hypersensitive patients with cystic fibrosis, the lymphocyte transformation test had a sensitivity of approximately 75% (36). In this study, three patients’ peripheral blood mononuclear cells were found to proliferate in the presence of sulfamethoxazole and/or its reactive nitroso metabolite. IFN‐γ PBMC ELISpot was used to confirm the positive result (**Supplementary Figure 2**). Two SMX and eleven SMX-NO specific T cells were generated from T-cell lines generated from two of the hypersensitive patients. Both patients carried HLA-B*13:01. The SMX-NO specific clones cross-reacted with the structurally related compound, nitroso dapsone (DDS-NO), which demonstrates the importance of the reactive nitroso functionality in the T cell response. A clinical cross-reactivity rate between co-trimoxazole and dapsone has been estimated to be approximately 22% and this may be due to the cross-reactivity of the metabolite-responsive T cells (37). Dapsone is related to SMX in that it contains an aromatic amine and a sulfone function, but the drugs differ in terms of their side-chains.

All of the SMX and SMX-NO specific T cell clones expressed a CD4+ phenotype which concordant to previous studies (38, 39). Immunohistochemical studies have shown that the cell infiltrate in maculopapular exanthema predominantly consists of CD4+ T cells (40, 41), whereas a predominance of epidermal CD8+ T cells is seen drug-induced bullous exanthem (42). Previous studies have revealed that drug-specific T cells secrete various cytokines including IFN-γ, IL-13, IL-22 (43, 44). The present study showed that both SMX and SMX-NO specific T cells secreted high levels of IL-13 along with lower levels of IL-22, IFN-γ and granzyme B secretion. However, IL-17 secretion from the clones was not observed. Eosinophilia is naturally reported in DRESS. Under inflammatory conditions, IL-13 is excreted by eosinophils which drives inflammatory responses and is typically associated with allergic inflammation (45–47).

Genetic association studies have shown a significant association between HLA-B*13:01 and co-trimoxazole-induced DRESS in patients with HIV infection (9). These data suggest that the causative drug might interact with the HLA-B*13:01 protein to activate CD8+ T cells in hypersensitive patients. Co-trimoxazole hypersensitivity is observed at a much lower frequency in patients without HIV infection. The reason for this is unclear, but may relate to a redox imbalance in patients with HIV infection, or altered metabolism, that leads to the formation of higher levels of sulfamethoxazole protein adducts (9, 48). A significant higher frequency of the HLA-B*13:01-C*03:04 haplotype was detected in co-trimoxazole-induced DRESS in the Thai population (9) and this is in linkage disequilibrium (LD) in Chinese (49) and Korean populations (50). Furthermore, these two alleles are also in LD with a HLA class II allele, namely HLA-DRB1*12:02 (50).

Somewhat surprisingly, the clones identified as drug-responsive in this study were CD4+ and T cell activation was diminished upon the blockade of *HLA* class II. This finding is in agreement with the study of Ogese and colleagues (38), which explored SMX T-cell responses in European patients, with hypersensitivity of mild to moderate severity, that were unlikely to express HLA-B*13:01. Ogese et al. demonstrated that the response of SMX-NO specific CD4+ T cells was restricted to the *HLA-DQ* allele, indicating that *HLA* class II plays an important role in the T cell activation in patients presenting with differing reaction phenotypes. In future studies it will be of interest to identify T cell receptor (TCR) repertoire expressed by drug-responsive T-cell clones and then determine their frequency in hypersensitive and tolerant patient PBMC.

The availability of SMX and SMX-NO responsive T cells allowed us to probe pathways of drug presentation by the HLA class II molecules. The SMX-NO responsive clones were stimulated to proliferate with APC pulsed with the drug metabolite for 1- and 16-hours. These data demonstrate that formation of a stable complex between the drug metabolite and antigen presenting cells is important for T cell activation. These data are concordant with Schnyder et al. which demonstrated the responsive T cell clones from SMX hypersensitive patients recognized covalently bound SMX-NO (39). The proliferative response of 5 out of 9 of the SMX-NO specific T cell clones analyzed was abolished APC were fixed with glutaraldehyde. This indicates that the T cell activation is dependent upon antigen processing and that the T cells are likely activated with drug-modified peptides. On the contrary, fixed antigen presenting cells had little effect on the activation of the remaining 4 clones. These clones are presumably activated when SMX-NO binds directly with surface peptides embedded within the HLA class II proteins. Finally, a SMX specific clone, SMX-43, was subjected to the same experiments. This clone was stimulated to proliferate with soluble drug in the presence of irradiated and fixed APC, while SMX-pulsed APC did not activate the T-cells. Direct interactions of drugs (p-i model) are not stable and washing the cells abolishes reactivity. Previous studies suggest that SMX may interact directly with either HLA-peptide complex (p-i HLA) or T cell receptors (p-i TCR) which can induce T cell activation (51, 52).

The tripeptide glutathione functions to prevent SMX-NO from covalently modifying proteins *via* quenching its reactivity (53, 54). Addition of glutathione to SMX-NO specific T cells blocked the induced proliferative response of the drug metabolite, whereas glutathione had no effect on the activation of clones with SMX (43, 53).

In conclusion, the generation of SMX and SMX-NO specific T-cell clones from co-trimoxazole hypersensitive patients suggests an immune mediated basis for the hypersensitivity reactions observed in individuals expressing HLA-B*13:01. The clones were CD4+ and activation was HLA class II-restricted indicating that HLA-B*13:01 was not directly involved in the disease pathogenesis.

## Data Availability Statement

The original contributions presented in the study are included in the article/**Supplementary Material**. Further inquiries can be directed to the corresponding author.

## Ethics Statement

The studies involving human participants were reviewed and approved by Institutional Review Boards in Mahidol University. The patients/participants provided their written informed consent to participate in this study.

## Author Contributions

TR, WT, NN, PR, JK and YS reviewed and collected the clinical data. CS, DN, and MP designed the research and supervised the project. JP, PT, KJ, RT, and PJ recruited subjects and performed the experiment. JP and PT analyzed the data and drafted the manuscript. CS, DN, and MP revised critical revision of the article and approved the final version to be published. All authors contributed to the article and approved the submitted version.

## Funding

This study was supported by grants from the (1) the Royal Golden Jubilee Ph.D. (RGJ-PHD) Program (PHD/0153/2559) (2) Newton Fund - PhD Placements for Scholars. (3) Huachiew Chalermprakiet University (4) The International Research Network-The Thailand Research Fund (IRN60W003) (5) Faculty of Medicine Ramathibodi Hospital, Mahidol University (6) Skin and Allergy Research Unit, Chulalongkorn University (7) Thailand Center of Excellence for Life Sciences (grant number TC-12/63) (8) Health System Research Institute under Genomics Thailand Strategic Fund (9) Merck Pharmaceuticals. The funder was not involved in the study design, collection, analysis, interpretation of data, the writing of this article or the decision to submit it for publication.

## Conflict of Interest

The authors declare that the research was conducted in the absence of any commercial or financial relationships that could be construed as a potential conflict of interest.

## Supplementary Material

Supplementary Figure 1 Flow chart of the study.

Supplementary Figure 2 The proliferative response and IFN-γ ELISPOT of three hypersensitive patients **(A)** hypersensitive patients’ PBMCs (1.5x10^6^) were cultures with SMX (1 and 2 mM), SMX-NO (10 and 20 µM), TMP (10 and 25 µM), DDS (125 and 250 µM) and DDS-NO (5 and 10 µM) for 6 days (37°C, 5% CO_2_). Following incubation, [3H]-thymidine (0.5 μCi) were added to measure proliferative response. **(B)** PBMCs (5x10^6^) were cultured in the presence of SMX (1mM), SMX-NO (20 µM), DDS (125 µM) and DDS-NO (10 µM) for 48 hours (37°C, 5% CO_2_). Following incubation, the plate was developed according to the manufactures instructions visualized by ELISpot AID reader.

Supplementary Figure 3 Cross-reactivity of SMX-NO specific T cell clones. Autologous EBV-transformed B-cells (1x10^4^) were incubated with SMX-NO specific clones (5x10^4^) in the presence of various drugs at difference concentration including SMX (1mM), SMX-NO (20 and 40 µM), DDS (62.5 and 125 µM), DDS-NO (5 and 10 µM) and phytohemagglutinin (PHA; 5 µg/mL). The proliferative response was measured using [3H]-thymidine incorporation assay.

## References

[1] Alanazi MQ Alqahtani FY Aleanizy FS. An Evaluation of E. Coli in Urinary Tract Infection in Emergency Department At KAMC in Riyadh, Saudi Arabia: Retrospective Study. Ann Clin Microbiol Antimicrob (2018) 17:3. 10.1186/s12941-018-0255-z 29422058 PMC5806437

[2] Ho JM Juurlink DN. Considerations When Prescribing Trimethoprim-Sulfamethoxazole. Cmaj (2011) 183:1851–8. 10.1503/cmaj.111152 PMC3216436 21989472

[3] Rueda-Valencia Mde L Infante S Campos M Beléndez C Lozano JS. Trimethoprim-Sulfamethoxazole-Induced DRESS Syndrome in a 4-Year-Old Child. Ann Allergy Asthma Immunol (2016) 116:366–7. 10.1016/j.anai.2015.12.009 26782674

[4] Taqi SA Zaki SA Nilofer AR Sami LB. Trimethoprim-Sulfamethoxazole-Induced Steven Johnson Syndrome in an HIV-infected Patient. Indian J Pharmacol (2012) 44:533–5. 10.4103/0253-7613.99346 PMC3469966 23087524

[5] Rosati E Dowds CM Liaskou E Henriksen EKK Karlsen TH Franke A. Overview of Methodologies for T-cell Receptor Repertoire Analysis. BMC Biotechnol (2017) 17:1–16. 10.1186/s12896-017-0379-9 28693542 PMC5504616

[6] Hausmann O Schnyder B Pichler WJ. Etiology and Pathogenesis of Adverse Drug Reactions. Chem Immunol Allergy (2012) 97:32–46. 10.1159/000335614 22613852

[7] Kongpan T Mahasirimongkol S Konyoung P Kanjanawart S Chumworathayi P Wichukchinda N. Candidate HLA Genes for Prediction of Co-Trimoxazole-Induced Severe Cutaneous Reactions. Pharmacogenet Genomics (2015) 25:402–11. 10.1097/FPC.0000000000000153 26086150

[8] Lonjou C Borot N Sekula P Ledger N Thomas L Halevy S. A European Study of HLA-B in Stevens-Johnson Syndrome and Toxic Epidermal Necrolysis Related to Five High-Risk Drugs. Pharmacogenet Genomics (2008) 18:99–107. 10.1097/FPC.0b013e3282f3ef9c 18192896

[9] Sukasem C Pratoomwun J Satapornpong P Klaewsongkram J Rerkpattanapipat T Rerknimitr P. Genetic Association of Co-Trimoxazole-Induced Severe Cutaneous Adverse Reactions is Phenotype-Specific: Hla Class I Genotypes and Haplotypes. Clin Pharmacol Ther (2020) 108:1078–89. 10.1002/cpt.1915 32452529

[10] Ko TM Chung WH Wei CY Shih HY Chen JK Lin CH. Shared and Restricted T-cell Receptor Use is Crucial for Carbamazepine-Induced Stevens-Johnson Syndrome. J Allergy Clin Immunol (2011) 128:1266–76.e11. 10.1016/j.jaci.2011.08.013 21924464

[11] Chung WH Pan RY Chu MT Chin SW Huang YL Wang WC. Oxypurinol-Specific T Cells Possess Preferential TCR Clonotypes and Express Granulysin in Allopurinol-Induced Severe Cutaneous Adverse Reactions. J Invest Dermatol (2015) 135:2237–48. 10.1038/jid.2015.165 25946710

[12] Pan RY Chu MT Wang CW Lee YS Lemonnier F Michels AW. Identification of Drug-Specific Public TCR Driving Severe Cutaneous Adverse Reactions. Nat Commun (2019) 10:3569. 10.1038/s41467-019-11396-2 31395875 PMC6687717

[13] Illing PT Vivian JP Dudek NL Kostenko L Chen Z Bharadwaj M. Immune Self-Reactivity Triggered by Drug-Modified HLA-peptide Repertoire. Nature (2012) 486:554–8. 10.1038/nature11147 22722860

[14] Yerly D Pompeu YA Schutte R Eriksson KK Strhyn A Bracey AW. Structural Elements Recognized by Abacavir-Induced T Cells. Int J Mol Sci (2017) 18:1464. 10.3390/ijms18071464 PMC5535955 28686208

[15] Bell CC Faulkner L Martinsson K Farrell J Alfirevic A Tugwood J. T-Cells From HLA-B*57:01+ Human Subjects are Activated With Abacavir Through Two Independent Pathways and Induce Cell Death by Multiple Mechanisms. Chem Res Toxicol (2013) 26:759–66. 10.1021/tx400060p 23541086

[16] Naranjo CA Busto U Sellers EM Sandor P Ruiz I Roberts EA. A Method for Estimating the Probability of Adverse Drug Reactions. Clin Pharmacol Ther (1981) 30:239–45. 10.1038/clpt.1981.154 7249508

[17] Sassolas B Haddad C Mockenhaupt M Dunant A Liss Y Bork K. ALDEN, an Algorithm for Assessment of Drug Causality in Stevens-Johnson Syndrome and Toxic Epidermal Necrolysis: Comparison With Case-Control Analysis. Clin Pharmacol Ther (2010) 88:60–8. 10.1038/clpt.2009.252 20375998

[18] Zhao Q Alhilali K Alzahrani A Almutairi M Juwaria A Liu H. Dapsone- and Nitroso Dapsone-Specific Activation of T Cells From Hypersensitive Patients Expressing the Risk Allele HLA-B*13:01. Allergy (2019) 74:1533–48. 10.1111/all.13769 PMC6767778 30844087

[19] Chen WT Wang CW Lu CW Chen CB Lee HE Hung SL. The Function of HLA-B*13:01 Involved in the Pathomechanism of Dapsone-Induced Severe Cutaneous Adverse Reactions. J Invest Dermatol (2018) 138:1546–54. 10.1016/j.jid.2018.02.004 29458119

[20] Tempark T Satapornpong P Rerknimitr P Nakkam N Saksit N Wattanakrai P. Dapsone-Induced Severe Cutaneous Adverse Drug Reactions are Strongly Linked With HLA-B*13: 01 Allele in the Thai Population. Pharmacogenet Genomics (2017) 27:429–37. 10.1097/FPC.0000000000000306 28885988

[21] Wang H Yan L Zhang G Chen X Yang J Li M. Association Between HLA-B*1301 and Dapsone-Induced Hypersensitivity Reactions Among Leprosy Patients in China. J Invest Dermatol (2013) 133:2642–44. 10.1038/jid.2013.192 23604100

[22] Hetherington S Hughes AR Mosteller M Shortino D Baker KL Spreen W. Genetic Variations in HLA-B Region and Hypersensitivity Reactions to Abacavir. Lancet (2002) 359:1121–2. 10.1016/S0140-6736(02)08158-8 11943262

[23] Mallal S Nolan D Witt C Masel G Martin AM Moore C. Association Between Presence of HLA-B*5701, Hla-DR7, and HLA-DQ3 and Hypersensitivity to HIV-1 Reverse-Transcriptase Inhibitor Abacavir. Lancet (2002) 359:727–32. 10.1016/s0140-6736(02)07873-x 11888582

[24] Locharernkul C Loplumlert J Limotai C Korkij W Desudchit T Tongkobpetch S. Carbamazepine and Phenytoin Induced Stevens-Johnson Syndrome is Associated With HLA-B*1502 Allele in Thai Population. Epilepsia (2008) 49:2087–91. 10.1111/j.1528-1167.2008.01719.x 18637831

[25] Tassaneeyakul W Tiamkao S Jantararoungtong T Chen P Lin SY Chen WH. Association Between HLA-B*1502 and Carbamazepine-Induced Severe Cutaneous Adverse Drug Reactions in a Thai Population. Epilepsia (2010) 51:926–30. 10.1111/j.1528-1167.2010.02533.x 20345939

[26] Sukasem C Chaichan C Nakkrut T Satapornpong P Jarutamsophon K Jantararoungtong T. Association Between HLA-B Alleles and Carbamazepine-Induced Maculopapular Exanthema and Severe Cutaneous Reactions in Thai Patients. J Immunol Res (2018) 2018:2780272. 10.1155/2018/2780272 29546073 PMC5818913

[27] Chung WH Hung SL Hong HS Hsih MS Yang LC Ho HC. A Marker for Stevens–Johnson Syndrome. Nature (2004) 428:486. 10.1038/428486a 15057820

[28] McCormack M Alfirevic A Bourgeois S Farrell JJ Kasperavičiūtė D Carrington M. Hla-A*3101 and Carbamazepine-Induced Hypersensitivity Reactions in Europeans. N Engl J Med (2011) 364:1134–43. 10.1056/NEJMoa1013297 PMC3113609 21428769

[29] Konvinse KC Trubiano JA Pavlos R James I Shaffer CM Bejan CA. Hla-A*32:01 is Strongly Associated With Vancomycin-Induced Drug Reaction With Eosinophilia and Systemic Symptoms. J Allergy Clin Immunol (2019) 144:183–92. 10.1016/j.jaci.2019.01.045 PMC6612297 30776417

[30] Sukasem C Jantararoungtong T Kuntawong P Puangpetch A Koomdee N Satapornpong P. Hla-B*58:01 for Allopurinol-Induced Cutaneous Adverse Drug Reactions: Implication for Clinical Interpretation in Thailand. Front Pharmacol (2016) 7:186. 10.3389/fphar.2016.00186 27486401 PMC4947582

[31] Hung SI Chung WH Liou LB Chu CC Lin M Huang HP. Hla-B*5801 Allele as a Genetic Marker for Severe Cutaneous Adverse Reactions Caused by Allopurinol. Proc Natl Acad Sci USA (2005) 102:4134–9. 10.1073/pnas.0409500102 PMC554812 15743917

[32] Tohkin M Kaniwa N Saito Y Sugiyama E Kurose K Nishikawa J. A Whole-Genome Association Study of Major Determinants for Allopurinol-Related Stevens-Johnson Syndrome and Toxic Epidermal Necrolysis in Japanese Patients. Pharmacogenomics J (2013) 13:60–9. 10.1038/tpj.2011.41 21912425

[33] Kano Y Hirahara K Mitsuyama Y Takahashi R Shiohara T. Utility of the Lymphocyte Transformation Test in the Diagnosis of Drug Sensitivity: Dependence on its Timing and the Type of Drug Eruption. Allergy (2007) 62:1439–44. 10.1111/j.1398-9995.2007.01553.x 17983378

[34] Tang YH Mockenhaupt M Henry A Bounoua M Naldi L Gouvello SL. Poor Relevance of a Lymphocyte Proliferation Assay in Lamotrigine-Induced Stevens-Johnson Syndrome or Toxic Epidermal Necrolysis. Clin Exp Allergy (2012) 42:248–54. 10.1111/j.1365-2222.2011.03875.x 22092454

[35] Naisbitt DJ Britschgi M Wong G Farrell J Depta JPH Chadwick DW. Hypersensitivity Reactions to Carbamazepine: Characterization of the Specificity, Phenotype, and Cytokine Profile of Drug-Specific T Cell Clones. Mol Pharmacol (2003) 63:732–41. 10.1124/mol.63.3.732 12606784

[36] Whitaker P Meng X Lavergne SN El-Ghaiesh S Monshi M Earnshaw C. Mass Spectrometric Characterization of Circulating and Functional Antigens Derived From Piperacillin in Patients With Cystic Fibrosis. J Immunol (2011) 187:200–11. 10.4049/jimmunol.1100647 PMC3145118 21606251

[37] Holtzer CD Flaherty JF Jr Coleman RL. Cross-Reactivity in HIV-infected Patients Switched From Trimethoprim-Sulfamethoxazole to Dapsone. Pharmacotherapy (1998) 18:831–5. 10.1002/j.1875-9114.1998.tb03904.x 9692656

[38] Ogese MO Saide K Faulkner L Whitaker P Peckham D Alfirevic A. Hla-DQ Allele-Restricted Activation of Nitroso Sulfamethoxazole-Specific CD4-Positive T Lymphocytes From Patients With Cystic Fibrosis. Clin Exp Allergy (2015) 45:1305–16. 10.1111/cea.12546 25851465

[39] Schnyder B Burkhart C Schnyder-Frutig K von Greyerz S Naisbitt DJ Pirmohamed M. Recognition of Sulfamethoxazole and its Reactive Metabolites by Drug-Specific CD4+ T Cells From Allergic Individuals. J Immunol (2000) 164:6647–54. 10.4049/jimmunol.164.12.6647 10843725

[40] Pichler WJ Yawalkar N Britschgi M Depta J Strasser I Schmid S. Cellular and Molecular Pathophysiology of Cutaneous Drug Reactions. Am J Clin Dermatol (2002) 3:229–38. 10.2165/00128071-200203040-00001 12010068

[41] Yawalkar N Egli F Hari Y Nievergelt H Braathen LR Pichler WJ. Infiltration of Cytotoxic T Cells in Drug-Induced Cutaneous Eruptions. Clin Exp Allergy (2000) 30:847–55. 10.1046/j.1365-2222.2000.00847.x 10848903

[42] Hertl M Jugert F Merk HF. CD8+ Dermal T Cells From a Sulphamethoxazole-Induced Bullous Exanthem Proliferate in Response to Drug-Modified Liver Microsomes. Br J Dermatol (1995) 132:215–20. 10.1111/j.1365-2133.1995.tb05016.x 7534104

[43] Elsheikh A Castrejon L Lavergne SN Whitaker P Monshi M Callan H. Enhanced Antigenicity Leads to Altered Immunogenicity in Sulfamethoxazole-Hypersensitive Patients With Cystic Fibrosis. J Allergy Clin Immunol (2011) 127:1543–51.e3. 10.1016/j.jaci.2010.12.1119 21354601

[44] Gibson A Ogese M Sullivan A Wang E Saide K Whitaker P. Negative Regulation by PD-L1 During Drug-Specific Priming of IL-22-Secreting T Cells and the Influence of PD-1 on Effector T Cell Function. J Immunol (2014) 192:2611–21. 10.4049/jimmunol.1302720 PMC3951492 24510967

[45] Mori F Caffarelli C Caimmi S Bottau P Liotti L Franceschini F. Drug Reaction With Eosinophilia and Systemic Symptoms (DRESS) in Children. Acta BioMed (2019) 90:66–79. 10.23750/abm.v90i3-S.8167 30830064 PMC6502175

[46] Schmid-Grendelmeier P Altznauer F Fischer B Bizer C Straumann A Menz G. Eosinophils Express Functional IL-13 in Eosinophilic Inflammatory Diseases. J Immunol (2002) 169:1021–7. 10.4049/jimmunol.169.2.1021 12097410

[47] Teraki Y Fukuda T. Skin-Homing Il-13-Producing T Cells Expand in the Circulation of Patients With Drug Rash With Eosinophilia and Systemic Symptoms. Dermatology (2017) 233:242–9. 10.1159/000475546 28601883

[48] Coco-Bassey SB Asemota EA Okoroiwu HU Etura JE Efiong EE Inyang IJ. Glutathione, Glutathione Peroxidase and Some Hematological Parameters of HIV-seropositive Subjects Attending Clinic in University of Calabar Teaching Hospital, Calabar, Nigeria. BMC Infect Dis (2019) 19:1–10. 10.1186/s12879-019-4562-6 31703562 PMC6842150

[49] Zhang FR Liu H Irwanto A Fu XA Li Y Yu GQ. Hla-B*13:01 and the Dapsone Hypersensitivity Syndrome. N Engl J Med (2013) 369:1620–8. 10.1056/NEJMoa. 1213096. 24152261

[50] Park HJ Park JW Kim SH Choi SY Kim HK Jung CG. The HLA-B*13:01 and the Dapsone Hypersensitivity Syndrome in Korean and Asian Populations: Genotype- and Meta-Analyses. Expert Opin Drug Saf (2020) 19:1349–56. 10.1080/14740338. 2020.1796965. 32700588

[51] Pacher P Nivorozhkin A Szabó C. Therapeutic Effects of Xanthine Oxidase Inhibitors: Renaissance Half a Century After the Discovery of Allopurinol. Pharmacol Rev (2006) 58:87–114. 10.1124/pr.58.1.6 16507884 PMC2233605

[52] Watkins S Pichler W. Activating Interactions of Sulfanilamides With T Cell Receptors. Open J Immunol (2013) 3:139–57. 10.4236/oji.2013.33019 PMC7613643 36172594

[53] Burkhart C von Greyerz S Depta JP Naisbitt DJ Britschgi M Park KB. Influence of Reduced Glutathione on the Proliferative Response of Sulfamethoxazole-Specific and Sulfamethoxazole-Metabolite-Specific Human CD4+ T-Cells. Br J Pharmacol (2001) 132:623–30. 10.1038/sj.bjp.0703845 PMC1572594 11159714

[54] Naisbitt DJ O’Neill PM Pirmohamed M Park BK. Synthesis and Reactions of Nitroso Sulphamethoxazole With Biological Nucleophiles: Implications for Immune Mediated Toxicity. Bioorganic Med Chem Lett (1996) 6:1511–6. 10.1016/S0960-894X(96)00260-0

