# Supplementary figures and images for: Characterization of T-Cell Responses to SMX and SMX-NO in Co-Trimoxazole Hypersensitivity Patients Expressing HLA-B*13:01
Source: Front Immunol. 2021 Apr 29;12:658593. doi: 10.3389/fimmu.2021.658593 (PMC8117787; doi:10.3389/fimmu.2021.658593)

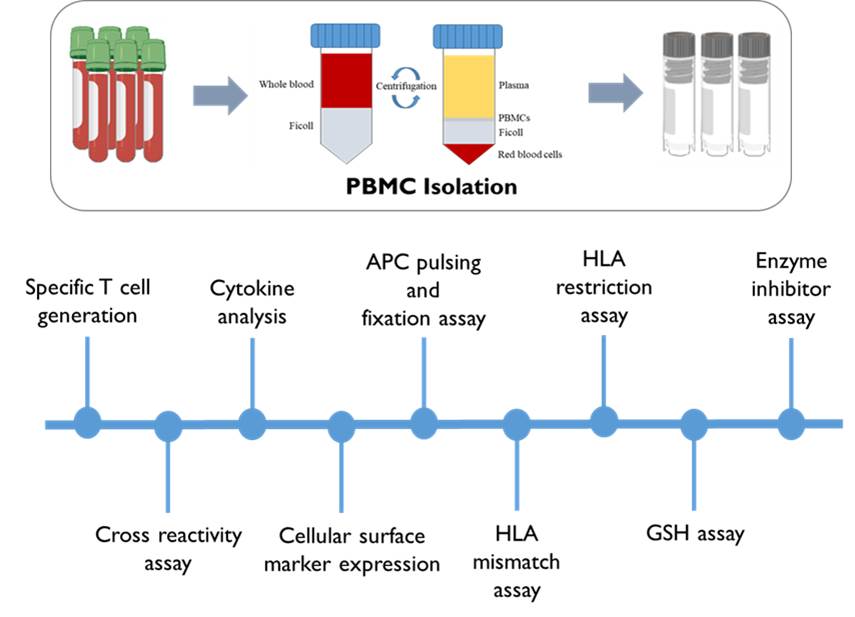

Supplement: Supplementary Figure 1 — Flow chart of the study. [file Image_1.jpg]

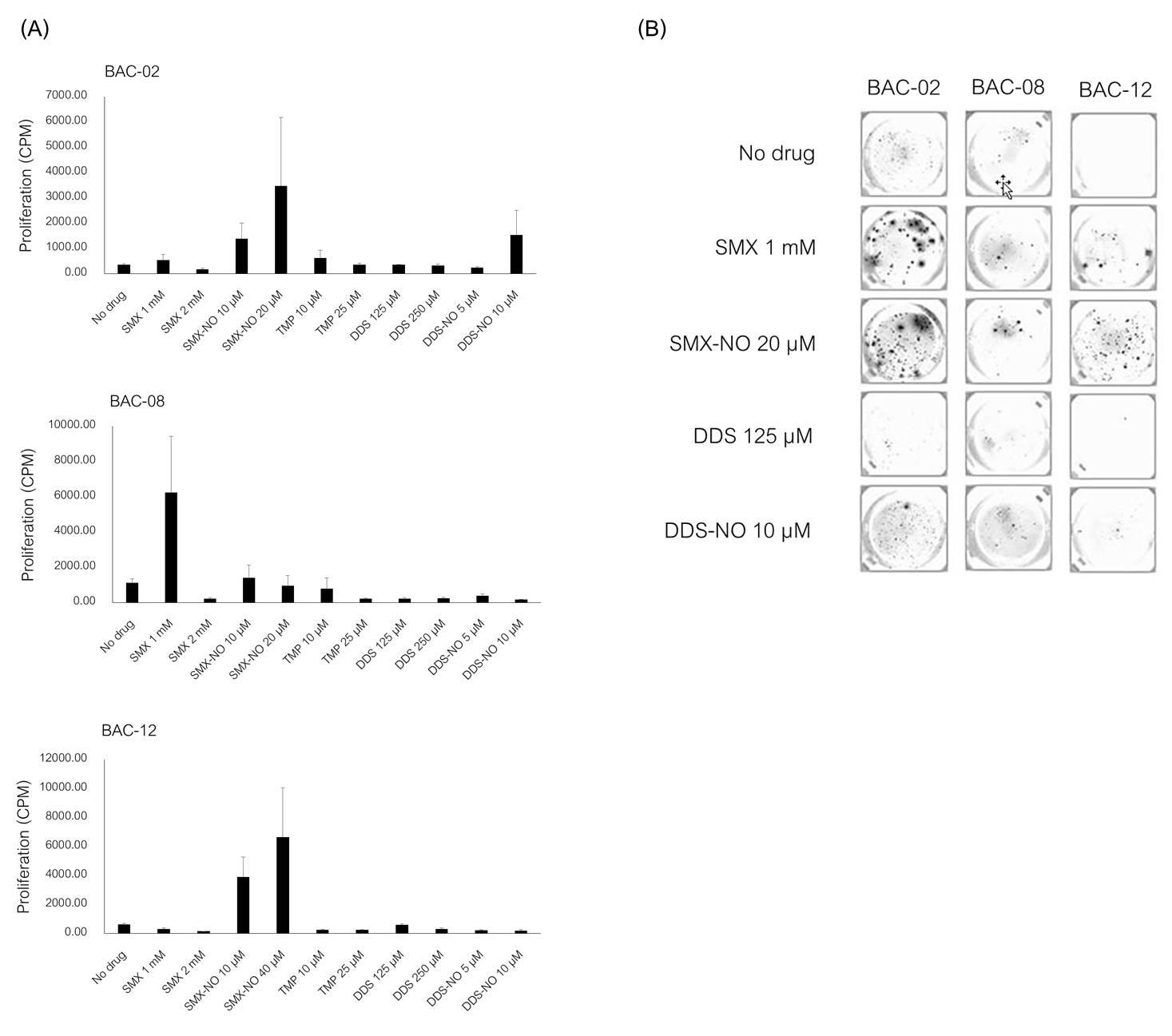

Supplement: Supplementary Figure 2 — The proliferative response and IFN-γ ELISPOT of three hypersensitive patients (A) hypersensitive patients’ PBMCs (1.5x10^6^) were cultures with SMX (1 and 2 mM), SMX-NO (10 and 20 µM), TMP (10 and 25 µM), DDS (125 and 250 µM) and DDS-NO (5 and 10 µM) for 6 days (37°C, 5% CO2). Following incubation, [3H]-thymidine (0.5 μCi) were added to measure proliferative response. (B) PBMCs (5x10^6^) were cultured in the presence of SMX (1mM), SMX-NO (20 µM), DDS (125 µM) and DDS-NO (10 µM) for 48 hours (37°C, 5% CO2). Following incubation, the plate was developed according to the manufactures instructions visualized by ELISpot AID reader. [file Image_2.jpg]

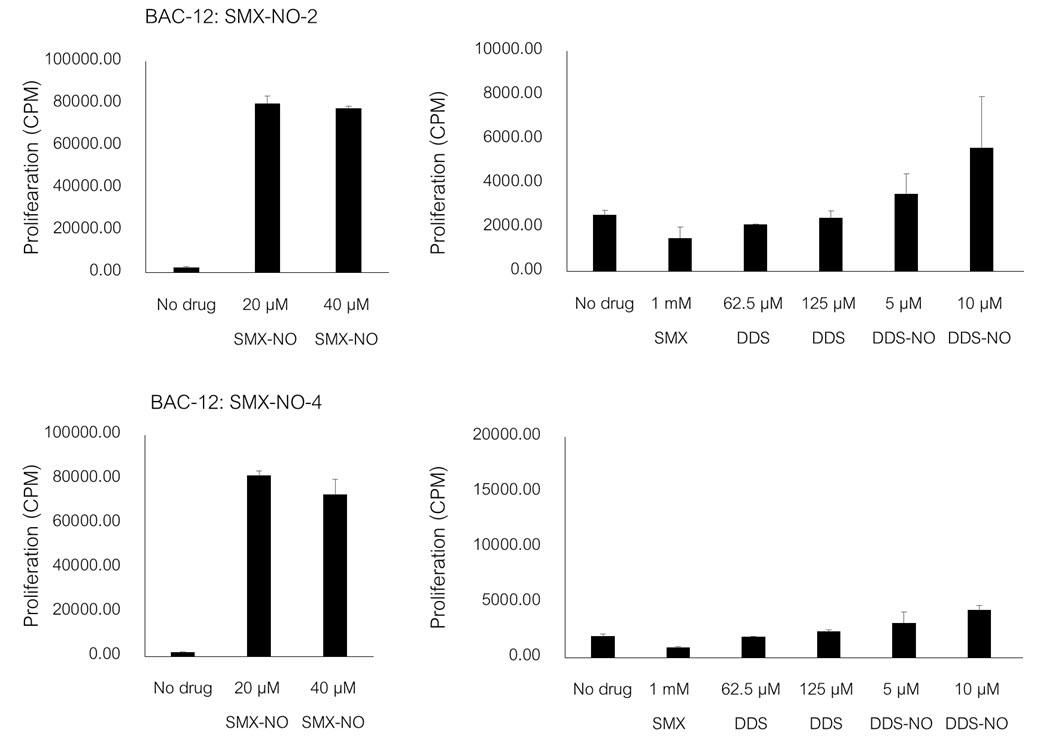

Supplement: Supplementary Figure 3 — Cross-reactivity of SMX-NO specific T cell clones. Autologous EBV-transformed B-cells (1x10^4^) were incubated with SMX-NO specific clones (5x10^4^) in the presence of various drugs at difference concentration including SMX (1mM), SMX-NO (20 and 40 µM), DDS (62.5 and 125 µM), DDS-NO (5 and 10 µM) and phytohemagglutinin (PHA; 5 µg/mL). The proliferative response was measured using [3H]-thymidine incorporation assay. [file Image_3.jpg]
