# Supplementary material for: Characterization of T-Cell Responses to SMX and SMX-NO in Co-Trimoxazole Hypersensitivity Patients Expressing HLA-B*13:01
Source: Front Immunol. 2021 Apr 29;12:658593. doi: 10.3389/fimmu.2021.658593 (PMC8117787; doi:10.3389/fimmu.2021.658593)
Supplement: Supplementary file 5 [file DataSheet_1.docx]

**Supplementary S1: Methods**

**T cell response analysis**

T cell response were tested by measurement of proliferation using [3H]-thymidine and cytokine secretion via ELISPOT. T cell clones (5x10^4^ cells/well) were incubated with irradiated autologous EBV-transformed B cells (1x10^4^ cells/well) in the presence and absence of the drugs or metabolites for a period of 48 hours (37°C, 5% CO_2_). Following incubation, 0.5μCi[3H]-thymidine were added and the cells were incubated for a further 16h. Next, the proliferation of T cells was measured by scintillation counting. T cell clones with a stimulation index (SI) (mean count per minute (cpm) drug treated wells/mean cpm in control wells) of greater than 2 were defined as positive response. Cytokine secretion was measured using ELIspot assay kits (Mabtech, Stockholm, Sweden). Briefly, 96-well polyvinylidene fluoride membrane plates were coated overnight at 4°C with coating antibody namely, anti-IFN-γ, -granzyme B, -IL-13, -IL-17 or -IL-22 antibody. Following overnight incubation, coated plates were washed five times with Hank's balanced salt solution (HBSS), blocked with R9 medium for 30 minutes at room temperature. Then, T cell clones (5x10^4^ cells/well) were incubated with irradiated autologous EBV-transformed B cells (1x10^4^ cells/well) in the presence and absence of the drugs or metabolites for a period of 48 hours (37°C, 5% CO_2_). Following incubation, the plates were washed with phosphate buffered saline (PBS) and biotinylated anti-cytokine detection antibody was added and incubated for 2 hours at room temperature, Next, plates were again washed and incubated with streptavidin–alkaline phosphatase for 1 hour at room temperature, then following washing, filtered BCIP/NBT substrate was added. Plates were incubated in the dark for spots to emerge. Spots were visualized using ELIspot AID reader.

**Generation and characterization of specific T cell clones**

Cell culture medium (R9) is composed of RPMI 1640 supplemented with 10% pooled human AB serum (Innovative Research, Class A), 25mM HEPES buffer, 2mM L-glutamine, 100 mg/ml streptomycin, 100U/ml penicillin and 25 mg/ml transferrin. T cell lines were generated by culturing PBMCs with dapsone (DDS, 125 µM), nitroso-dapsone (DDS-NO, 10 µM), sulfamethoxazole (SMX, 1 mM) and nitroso-sulfamethoxazole (SMX-NO, 20 µM)in R9 medium for 14 days (37°C; 5% CO_2_) and R9 media containing IL-2 (Concentration) was added to maintain proliferation on day 6 and 9. T cells clones were generated by serial dilution. Briefly, a serial dilution cocktail for each condition (0.3, 1 and 3 cells/well) was prepared by adding irradiated healthy donor PBMC (5x10^4^ cells/well), phytohemagglutinin (PHA) (5µg/ml), IL-2 (2µl/ml). After that, cells were plated in a 96 well U-bottomed plate and cultured for 14 days. The cells were restimulated with healthy donor’s APC mismatched PBMC after 14 days and then incubated for another 14 days. During that time well-growing wells were picked and split across four wells in a new 96 well plate.

Testing of T cell clones for drug specificity, well-growing clones were tested for drug specificity via analysis of proliferation in the presence of the drugs or metabolites. Two wells of T cell clones (5x10^4^ cells/well) were mixed and split across four new wells on a fresh 96 well plate and autologous EBVs (1x10^4^ cells/well) were added. Drug/metabolite was added to two of the wells and R9 media was added to the other two as a negative control. The cells were incubated for 48 hours. Proliferation was measured by the addition of [3H] thymidine for followed by an additional 16-hourincubation, before proliferation was measured using scintillation counting. Clones with a stimulation index (SI) (mean counts per minute (cpm) drug treated wells/mean cpm in control wells) of greater than 2 were expanded and analyzed further.

**Cellular surface marker expression**

FITC-labelled CD4 and PE-labelled CD8 were used for cellular surface marker expression which was assayed using flow cytometry. T cell clones (50 µL) were transferred to FACS tube and stained with 3µL of CD4-FITC and 0.5µL of CD8-PE and incubated on ice in the dark for 20 mins. Next cells were washed via centrifugation for 6 mins 1500 rpm at 4^o^C and resuspended in FACS buffer. Cells were analyzed on FACScantoII.

**HLA mismatch assay**

SMX-NO specific T cell clones were cultured with allogenic EBVs carrying *HLA-B*13:01*, *HLA-B*57:01* and other alleles in the presence or absence of 40 µM nitroso sulfamethoxazole for 48 hours (37°C, 5% CO_2_). Finally, [3H]-thymidine was added for another 16 hours of experiments. The proliferation of T cells was measured by scintillation counting

**HLA restriction assay**

Autologous EBV-transformed B cell lines were incubated with either 5 μg/ml anti-human HLA class I and HLA class II antibodies (BD Bioscience, Oxford, UK) for 30 minutes (37°C, 5% CO_2_), then co-cultured with specific T cell clones with or without drug antigen (40 μM SMX-NO) for 48 hours (37°C, 5% CO_2_) Following incubation, 0.5 μCi [3H]-thymidine was added and the cells were incubated for a further 16h and T cell responses were analyzed as previously described.

**Pathways of T cell activation in response to antigen stimulation**

For antigen presenting cell pulsing and fixation assay, specific T cell clones were cultured with 1 or 16h drug/metabolite-pulsed irradiated autologous EBV-transformed B cells in the absence of soluble drug for 48 hours (37°C, 5% CO_2_). EBV-transformed B cells were incubated with the 1 mM sulfamethoxazole or 40 µM nitroso sulfamethoxazole for 1 and 16 hours, then washed cell extensively and co-cultured with drug-specific T cell clones for 48 hours (37°C, 5% CO_2_). For APC fixation assay, glutaraldehyde (25%, 2 µL) was added to fix EBV-transformed B cells followed by immediate addition of glycine (1M, 1 mL). Next, cells were washed extensively were added with HBSS and re-suspended in R9 medium. Subsequently, drug-specific T cell clones were incubated with glutaraldehyde-fixed EBV-transformed B cells in the presence or absence of 1 mM sulfamethoxazole or 40 µM nitroso sulfamethoxazole for 48 hours (37°C, 5% CO_2_). Following incubation, 0.5 μCi [3H]-thymidine were added and the cells were incubated for a further 16h and T lymphocyte proliferation response were evaluated using scintillation counting.

To assess the effect of quenching on T cell responses, reduced glutathione (1 mM) was pre-cultured with autologous EBV-transformed B cells in the presence or absence of 1 mM SMX or 40 µM SMX-NO for 2 hours (37°C, 5% CO_2_). After 2 h incubation, the measure of T cell response was then conducted as previously described.

For T cell activation in response to the drugs in the presence of drug-metabolizing enzyme inhibitors, the experiment was conducted in the presence of drug-metabolizing enzyme inhibitors, methimazole (an inhibitor of peroxidases and flavin–mono-oxygenases; Meth) and 1-aminobenzotriazole (a nonselective suicide inhibitor; ABT), at a concentration that blocks SMX metabolism (1 mM) using IL-13 ELIspot as previously described.
